# Determination of the Dielectric Constant of Niobium Oxide by Using Combined EIS and Ellipsometric Methods

**DOI:** 10.3390/ma16020798

**Published:** 2023-01-13

**Authors:** Krzysztof Fitzner, Michał Stępień

**Affiliations:** Faculty of Non-Ferrous Metals, AGH University of Science and Technology, al. A. Mickiewicza 30, 30-059 Krakow, Poland

**Keywords:** niobium oxide, dielectric constant, ellipsometry, electrochemical impedance spectroscopy

## Abstract

Combining ellipsometric and EIS methods, the dielectric constant ε for the oxide Nb_2_O_5_ at room temperature was determined. At first, the linear dependence between anodization voltage and oxide thickness was established in the form *d* = 2.14 (± 0.05) · *U* + 12.2 (± 1.7) nm in the range of anodizing potentials 0–50 V. Next, assuming the equivalent circuit corresponds to one, the capacitance *C* of the dense oxide layer was measured. All results taken together gave the value of dielectric constant ε = 93 ± 5.

## 1. Introduction

Electrolytic capacitors are widely used in all kinds of consumer electronics. They can be found in computer power supplies, motherboards, amplifiers, electric motor controllers and telecommunications devices. The production of electrolytic capacitors, and more precisely their anodes covered with a dielectric layer, is based on three metals: aluminum, tantalum and niobium [1,2]. Tantalum-based capacitors are characterized by high capacity and also high price, while those based on aluminum are cheap but do not have such good performance parameters. Niobium exhibits similar chemical properties to tantalum; thus, it is not surprising that it is the first choice to substitute tantalum-based capacitors. Its better accessibility and lower cost resulted in extensive efforts to develop niobium capacitor technology. Niobium oxide is an n-type semiconductor with a band gap of ~3.4 eV and, as it is a transparent dielectric material, it is ideal for capacitor technology. The value of the dielectric constant ε given in the literature is 41 [2,3]. One of the first descriptions of niobium oxide film growth in aqueous acidic solution was given by Young [4]. Impedance measurements of the capacity of the oxide film were used for the determination of the film thickness (*d*). These were performed under the assumption that the resistance of the electrode is a linear function of this thickness. No value of the dielectric constant ε was mentioned in this work. Fuschillo et al. [5] presented Cole–Cole plots for films obtained after anodization, which was conducted in organic solutions at 100 °C. Thick, amorphous Nb_2_O_5_ layers were obtained. Despite the fact that capacitance and resistance were measured as a function of frequency, again no value of ε was reported. Later, Gomes et al. [6] coupled electrochemical techniques with ellipsometry. They found that diffusion of H^+^ ions into oxide films took place. The ratio of Nb/O atoms measured using ESCA (XPS) indicated enrichment in O atoms at the surface of the film. Thus, one may expect a change in Nb valency across the film thickness. It seems that the simultaneous application of electrochemical techniques and ellipsometry enhances the chance to obtain better descriptions of the film properties. However, accumulated experimental evidence indicates that it is difficult to extract the right value of the dielectric constant from EIS (electrochemical impedance spectroscopy) measurements since obtained 1/*C* vs. *d* dependence is nonlinear, and it also depends on the range of applied potentials. Such a change in the slope was demonstrated for anodic oxide films on tantalum by Kerrec et al. [7]. More extensive study of oxide film formation was carried out by Cavigliasso et al. [8] on tantalum and niobium. They investigated the influence of the forming electrolyte on the oxide film, which was next characterized using EIS. These experiments lead to the determination of the dielectric constant ε, which, depending on the electrolyte, varied from ~50 to ~120. Their work confirmed previous observations concerning 1/*C* vs. *d* dependence, but the oxide layer thickness was calculated from using Faraday’s law.

The advantage of the application of ellipsometry to the characterization of a thin film was demonstrated by Colard [9], who demonstrated the connection between refractive index and the thickness and homogeneity of the film grown on the surface. Graca et al. [10] made a distinction between various niobium oxides and concluded that the film of Nb_2_O_5_ is the most stable one. Depending on temperature, it may evolve from an amorphous state to monoclinic structure as the temperature rises. Ellipsometric study of passive and anodic oxide films on Nb and Ti was conducted by Arsov and Mickova [11,12] While ellipsometry provided the thickness of the passive layer, anodic oxidation can lead to controlled thickening of the oxide. Potentiodynamic studies found that Ti and Nb passive films exhibit different behavior, and Nb film is more resistant in acidic solutions than Ti. Recently, Komatsu et al. [13] investigated the color change mechanism of niobium oxide film in relation to its thickness. They confirmed that ellipsometry is a reliable tool to determine the thickness. The Nb_2_O_5_ film was produced via metal anodization. Its thickness was measured, and the influence of its variations on the color change was observed. This very useful dependence allows for control of the thickness of the oxide layer during electrolytic capacitor production without using any sophisticated tools.

Currently, the main trend in the development of electrolytic capacitors is to increase the product CV per unit of mass or volume [2]. Assuming that the capacitor operating voltage is close to the dielectric anodizing voltage, one will pay for the increase in operating voltage with a decrease in capacity *C* by increasing the thickness of the dielectric material. The thickness *d* is generally described by the relationship *d* = α · *U* where α is the anodization coefficient (slope of *d* vs *U* function) (V/nm). One can also try to increase the capacity by increasing the dielectric constant, whose value is accepted as 41. However, some results show that the dielectric constant for niobium may be around 120 [8] and, like for tantalum, may change with anodizing voltage and the electrolyte [7].

Therefore, it was decided that in order to accurately determine the dielectric constant of anodic oxide film on niobium, we need to use independent measurements of dielectric capacity and the thickness. Consequently, ellipsometry and EIS were both applied in this work. 

## 2. Materials and Methods

**Figure 1:**
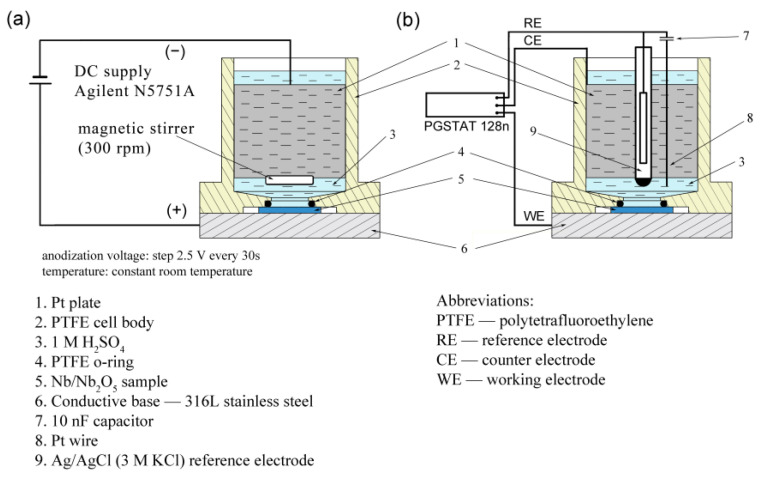
Electrochemical cell setup scheme: (**a**) anodizing samples, (**b**) EIS measurements.

Oxide thin films of Nb_2_O_5_ were prepared via anodization of Nb foils, 2 mm thick, containing 99.8% (Alfa Aesar, Karlsruhe, Germany) niobium. The plates were mechanically polished to a mirror finish (up to 0.05 µm Al_2_O_3_), thoroughly washed and ultrasonically cleaned in ethanol after every operation. Anodization was conducted in a solution of 1 M H_2_SO_4_ (Avantor Performance Materials Poland SA, Gliwice, Poland), with the solution magnetically stirred (300 rpm). The anodization voltage was increased by step 2.5 V every 30 s until a final voltage was obtained [12]. After reaching the final value, the voltage was held for 30 s, then the cell was turned off. For this voltage range, a two-electrode system was used with an Agilent N5751A DC power supply (Santa Clara, CA, USA) and platinum plate as the cathode (cell setup Figure 1a). In some cases, the anodization current was measured using a Keithley 2000 (Cleveland, OH, USA) digital multimeter. After anodization, the samples were gently flushed with deionized water and ethanol, and next the samples were dried in a warm air stream (approx. 50 °C).

Electrochemical impedance spectroscopy (EIS) measurements were carried out on the potentiostat AUTOLAB PGSTAT 128n (Utrecht, Netherlands) with a FRA2 module. The measurements were performed at room temperature using a three-electrode cell holding 30 ml of 1 M H_2_SO_4_ electrolyte with the diameter of the exposed oxide surface equal to 8 mm. An Ag/AgCl reference electrode with parallel Pt wire via 10 nF capacitor was used, while the counter-electrode was a platinum plate (cell setup Figure 1b). A frequency range from 0.05 Hz to 100 kHz was used and the amplitude of the voltage modulation was 30 mV. Schematic cell arrangements are shown in Figure 1a and 1b. The obtained EIS spectra were fitted to a chosen equivalent-circuit model with Z-view Software.

**Figure 2:**
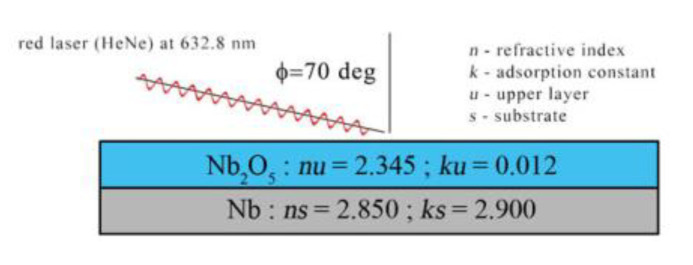
Niobium oxide model for ellipsometric measurements.

Ellipsometric measurement were performed using a SENTECH SE400adv ellipsometer (Berlin, Germany), choosing an angle of incidence of 70°. The example ellipsometric parameters *nu*, *ku*, *ns* and *ks* are presented in Figure 2 and were taken from the work of Arsova kloi0p9,et al. [12].

The surface of the obtained samples was analyzed using a JEOL JCM7000 scanning electron microscope (SEM) (Tokyo, Japan).

The X-ray photoelectron spectra (XPS) were recorded using the hemispherical analyzer EA 15 (PREVAC, Rogów, Poland) equipped with dual anode X-ray source RS 40B1 (PREVAC). The measurements were performed using Al Kα (1486.7 eV) radiation and an analyzer pass energy of 100 eV. The spectra were recorded in normal emission geometry with an energy resolution of 1.0 eV. The spectrometer was calibrated with Ag, Au and Cu foil according to ISO 15472:2010 standard. Ultra-high vacuum (UHV) conditions of 1 · 10^−9^ mbar were maintained during the measurements. The area of analysis was approximately 3 mm^2^ and the depth of analysis was about 10 nm. 

## 3. Results

### 3.1. Ellipsometric Measurements

In order to measure oxide layer thickness using ellipsometry, optical constants for the adopted model must be known (complex refractive index of the substrate metal, the oxide film and the surrounding medium). These values depend not only on the type of a material, but also on the surface preparation method. In the case of the oxide film obtained on niobium via the electrochemical oxidation method, a one layer-model is assumed [12,14]. This one-layer model, shown in Figure 2, was used in this work in all evaluations of ellipsometric data with various optical constants. However, it should be pointed out that a two-layer model has been also analyzed [15]. The optical constants for Nb and Nb_2_O_5_ found in the literature [12,16,17] show big discrepancies. Different values of optical constants for the substrate layer result mainly from the method of surface preparation, mechanical polishing or mechanical polishing combined with finishing electropolishing (hereinafter abbreviated as electropolishing). On the other hand, the optical constant for the oxide film results mainly from the measurement conditions (in situ or ex situ); however, influence from the substrate preparation method is also possible [12,16]. In this work, the samples were mechanically polished and the measurements were made in ex situ conditions. Unfortunately, it was not possible to find in the literature exactly such a combination of optical constants that would give reasonable measurement results. Therefore, in this paper the various optical constant sets were taken into account. The proposed optical constant sets used as models are shown in Table 1.

**Figure 3:**
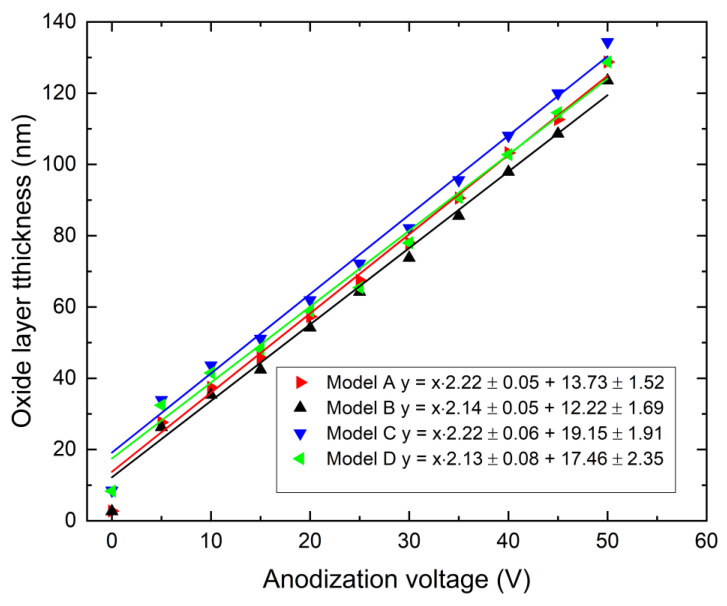
Ellipsometric measurements of oxide film growth in 1 M H_2_SO_4_ on mechanically polished Nb anode vs. applied voltage for various optical constants shown in Table 1.

For the chosen model version, ellipsometric measurements were performed. Measured samples were anodized with voltages changing from 0 to 50 volts (0 volts means that measurements were performed after polishing). The obtained results for various optical constant sets are shown in Figure 3. Error bars for obtained oxide thickness are not marked in Figure 3 to improve readability. In all cases, uncertainties of type A [18] calculated for significance level 0.05 and number of observations 15 (three samples for one anodization voltage and five points for each sample) do not exceed 1 nm. The obtained dependencies are linear.

The coefficients of film thickness growth and the thickness of the initial oxide film were calculated using linear regression (calculations were made without the 0 voltage point) and are presented in Table 2, while the results are shown in Figure 3. It can be seen that these results do not show significant difference. For a non-anodized sample (0 V), the difference in thickness of the oxide film obtained from regression and measured by ellipsometry is equal approximately to 10 nm, no matter which model was used. This initial oxide layer is the result of the fact that the samples were in contact with the electrolyte, anodization voltage was not equal to the reference potential, and drying of specimens was conducted in warm air. Moreover, aging phenomena might have also occurred [12].

For further considerations, we chose model B, since the evaluations of angle deviation errors did not exceed 3° (this is a measurement validation criterion built into the ellipsometer software) in the whole voltage range, and the initial oxide layer thickness agreed with the majority of other studies.

### 3.2. Impedance Measurements

**Figure 4:**
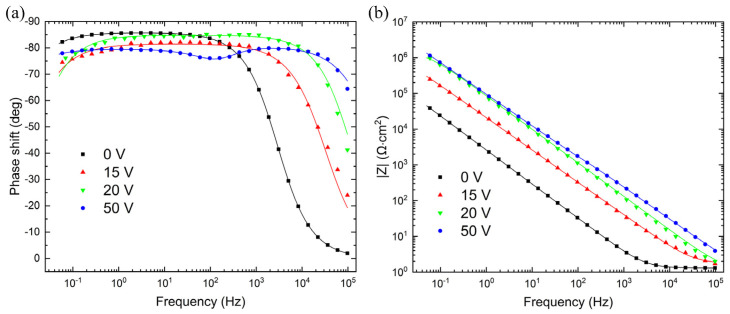
Impedance spectra for selected oxide films grown with different anodization voltage, obtained in 1.0 M H_2_SO_4_. (**a**) Phase shift, (**b**) magnitude of the impedance.

Figure 4 shows the selected impedance spectra presented as a Bode plot ((Figure 4a) phase shift, (Figure 4b) magnitude) for oxides grown in 1.0 M H_2_SO_4_ under an anodization voltage varying from 0 to 50 V. Points represent measurement results and continuous curves correspond to obtained fits with Z-View software. Due to measurements of the thickness of the niobium oxide layer via ellipsometry, its dielectric constant *ε* can now be determined using the EIS method. The parallel-plate condenser equation in the form:

(1) $$C=\frac{\varepsilon_{0}\cdot \varepsilon \cdot A\cdot r}{d}$$

was used. *A* is the geometric area, *d* is the thickness of the oxide film (obtained via ellipsometric measurements), *r* is the roughness factor equal to 1 (this is the typically accepted value in the literature for mechanically polished samples [8]), *ε* is the dielectric constant, and *ε*_0_ = 8.85 · 10^−12^ F/m. The capacity of the oxide layer was calculated from EIS spectra with the fitted equivalent circuit.

The simplest way to determinate the capacity is to adopt the ideal capacitor model and to use a single-frequency measurement [19]. In such a case, the capacity is obtained from the Equation (2):

(2) −Im(Z)=1ω·C

where chosen frequency *f* is equal 972 Hz (to avoid harmonic contribution from the power supply frequency 50 Hz). This value is similar to that used by other researchers (1 kHz [4]). Moreover, if the chosen frequency (which is to be measured) is sufficiently high (ω >> *RC*), we do not make an error in estimating capacitance, even if the parallel resistance exists. This error may appear for a system where the capacity is described as CPE, with *n* parameter different from 1. However, the widely used theoretical model for metal oxide–electrolyte systems is the R(RC) circuit. The oxide layer in this model is assumed to be an ideally homogenous one. In our case, due to surface inhomogeneity, the aforementioned model gave poor fitting results. A more reasonable approach is to consider the oxide as a non-homogenous layer and replace the capacitance (*C*) with a constant phase element (CPE) [19,20,21].

(3) $$Z(\omega )=\frac{1}{C\cdot {\left(j\cdot \omega \right)}^{n}}$$

**Figure 5:**
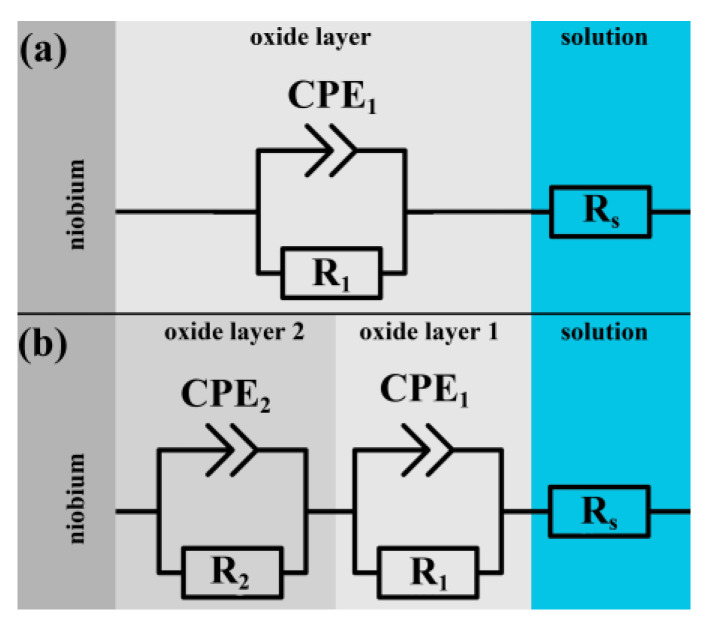
Used equivalent circuits for EIS measurements (**a**) single layer, (**b**) double layer.

The obtained equivalent circuit with CPE is shown in Figure 5a. However, more accurate examination of the phase angle vs. log *f* curve reveals a second time constant (e.g. in Figure 4, for 50 V a center of inflection is observed at about 100 Hz). For this reason, it seems more reasonable to use the two-layer model (shown in Figure 5b) for the oxide obtained for higher anodization voltage. Then, the transfer functions in previously discussed equivalent circuits are given by Equations (4) and (5):

(4) $$Z(\omega )=R_{s}+\frac{R_{1}}{1+R_{1}\cdot C_{1}\cdot {\left(j\omega \right)}^{n}}$$

(5) $$Z(\omega )=R_{s}+\frac{R_{1}}{R_{1}\cdot C_{1}\cdot {\left(j\omega \right)}^{n_{1}}}+\frac{R_{2}}{R_{2}\cdot C_{2}\cdot {\left(j\omega \right)}^{n_{2}}}$$

for the single- (Figure 5a) and two-layer model (Figure 5b), respectively. *R*_x_ and *C*_x_ represent the oxide layer resistance and capacitance, respectively, *R*_s_ is the solution resistance, *j* = √(−1), and ω = 2π*f*—is the angular frequency. All three models were tested in this work.

The oxide layer capacitance *C* and parameter *n* gathered in Table 3 represent the CNLS (complex nonlinear least squares) fit of the data to Equations (4) and (5) while *C* was also obtained directly from Equation (2). A single-layer model, whose impedance is given by Equation (4), returns good fit of results for the oxide layer obtained in the anodizing voltage up to 30 V. For anodizing voltages above 30 volts, the model that takes into account two non-homogenous layers was used. In this case, satisfactory values of error and residues for CNLS fit were obtained. However, the obtained capacitance values were too large even for a double-layer capacity or space charge (expected value 20–40 µF/cm^2^ [22]). This result can indicate the appearance of cracks in the oxide film layer [4]. Moreover, we were not able to estimate the thickness of individual layers in this case, because we do not know the dielectric constant. The shaded fields in Table 3 show the cases for which the use of the selected EIS model was pointless due to large values of errors and residuals.

**Figure 6:**
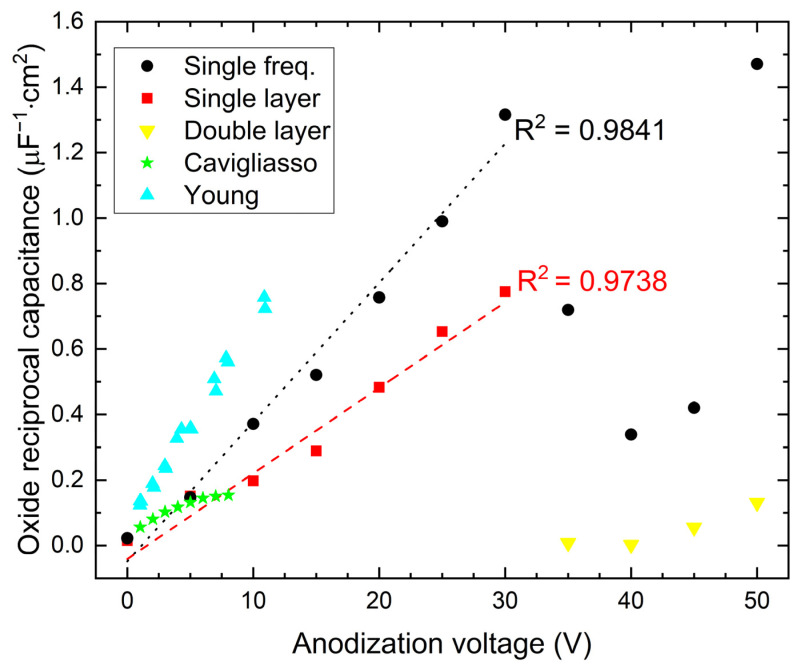
Oxide reciprocal capacitance as a function of anodization voltage for Nb oxide films.

The alternative estimate of the oxide film capacity based on EIS was performed in this work with the use of a single frequency. It seems to underestimate obtained values as compared to measurements using the entire EIS spectrum. This tendency may be due to the inhomogeneity of the obtained oxide film. The obtained value of the *n* parameter had a mean value of about 0.95 and less. After all, this method gives a linear dependence of reciprocal capacity on forming voltage (see Figure 6). The obtained reciprocal capacity was slightly smaller than that obtained under similar conditions by Young [4].

The dielectric constant of Nb_2_O_5_ calculated from the measurements of thickness and capacity of the oxide films is shown in Table 4. Row A shows the calculated values for capacitance obtained with a single frequency, row B for impedance given by Equation (4). Due to the difference between the initial thickness of the oxide obtained via linear regression and measured using ellipsometry, for calculations of the dielectric constant for 0 V, the initial oxide thickness was assumed to be 3 nm. The mean value of the dielectric constant ε was calculated from the slope of the plot reciprocal capacitance as the function of applied voltage. For the capacity obtained from the EIS single-layer model, the value of the dielectric constant was 93 ± 5, while for single-frequency measurements, the dielectric constant was equal to 57 ± 4 (error calculated directly via line fits). Error calculated via propagation of uncertainty in the worst-case scenario should not exceed 34% (assuming estimation error *d* is equal to 14% and error for *C* is equal to 20%). The obtained values of ε were higher than those derived from different models of EIS interpretation reported in the literature.

Graca et al. [10] obtained the lowest values of the Nb_2_O_5_ dielectric constant, in the range of 8–16. They synthesized the oxide film using the DC reactive sputtering method and carried out their measurements in helium atmosphere at high frequencies (100 kHz). Similar measurements were made by Cavigliasso et al. [8]. They gave the value of the dielectric constant of Nb_2_O_5_ around 119 ± 7, but only for the formation potential in the range of 0–4 V (even though the formation potential were examined up to 8 V). Analyzing the data from their work for the anodizing potentials between 5 and 8 volts, it is clear that the dielectric constant of Nb_2_O_5_ must be significantly greater than 120.

### 3.3. SEM Observation

**Figure 7:**
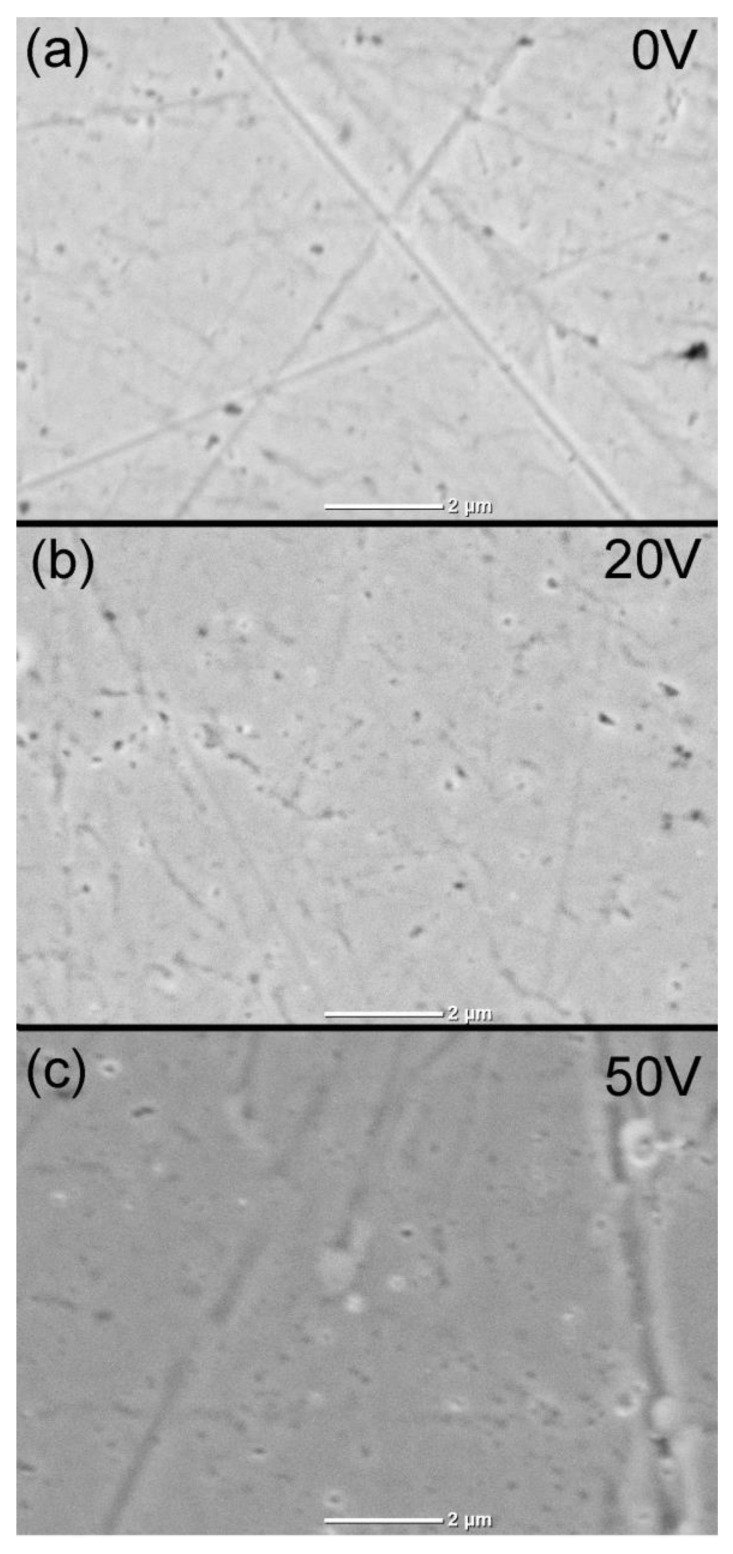
SEM image of the sample surface at (**a**) 0 V before anodization, (**b**) 20 V and (**c**) 50 V after anodization.

The results of the sample morphology tests using SEM are shown in Figure 7. On the surface of the non-anodized sample (Figure 7a, 0 V), scratches formed during the sample polishing stage are clearly visible. The tiny black spots on the surface are most likely polishing remnants that could not be removed during the ultrasonic cleaning procedure. After anodization of the metal surface, small pits and wormlike holes (Figure 7b, 20 V and Figure 7c, 50 V) appeared. This is probably due to the internal stress created during oxide growth. Volume expansion and electrostrictions processes are responsible for the formation of these stresses during anodization [23]. The size of these structures increases with increasing anodizing voltage. These pits and holes may build another capacitor in series, which greatly reduces the total capacity (see Figure 6, anodization voltage above 30 V). Moreover, two time constants on Bode plots suggest two-layer structure.

### 3.4. XPS Measurements

**Figure 8:**
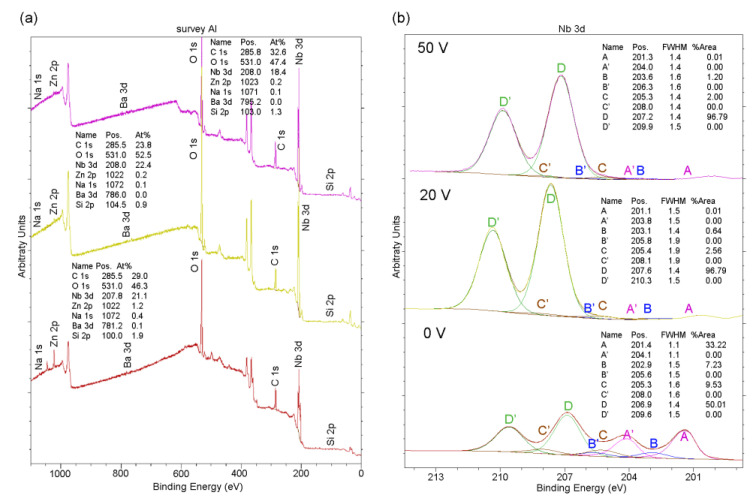
XPS (**a**) spectra for Nb anodized 0 V (bottom), 20 V and 50 V (top), (**b**) Nb 3d spectra.

The XPS spectra of the samples analyzed using SEM were recorded and are shown in Figure 8. Niobium on the surfaces of all anodized samples occurred in the form of Nb_2_O_5_ (peak D). For not-anodized samples, niobium occurred as Nb_2_O_5_, NbO_2_ (peak C), NbO (peak B) and metallic Nb (peak A). Oxygen on the surfaces of all samples occurred mainly in the oxide Nb_2_O_5_, but also in some compounds formed together with other elements on the surface layer. Carbon on the surfaces (Figure 8a, C 1s spectra) of all samples occurred mainly as hydrocarbon, which can be considered contamination due to the sample preparation procedure.

## 4. Discussion

Over the last 50 years, a lot of work has been performed on the dielectric properties of the oxide layer on niobium. The obtained results were frequently inconsistent. For example, the measured dielectric constant of Nb_2_O_5_ has a value between 6 and 16 as reported by Graca et al. [10], while in the work [8] it is equal to 120. The value of constant ε seems to depend on measurement methods and the synthesis process. In all works cited in this paper, to calculate the dielectric constant of Nb_2_O_5_, the oxide thickness must be known. In the cases when the oxide layer is obtained using an electrochemical method, a good way to estimate the oxide thickness is to use the anodization coefficient α. The obtained results show that the oxide thickness depends linearly on the voltage/potential regardless of the type of the electrolyte [8,12,13]. This allows the anodizing coefficient to be compared as a substitute for real oxide thickness. Table 5 shows anodization coefficients gathered from different sources.

The results of ellipsometric measurements depend on the optical constants used during their interpretation [12]. In this work, we used optical constants accepted from the literature. They differ, however; therefore, we made use of several available models (see Table 1). Assuming that the dependence *d* = f(*U*) is linear [12], and accepting as a criterion of this linear dependence the coefficient of determination R^2^ at the level 0.99+, all models we used were linear. For this check, the results for samples denoted as V = 0 were not included in our calculations. The obtained anodization coefficients α for all models are similar and agree well with the literature (Table 5). However, large differences in the values found in the literature as well as among models can be noticed for the refraction coefficient of niobium deprived of the oxide layer. This may be caused by the difficulties in the measurements connected with either fast passivation of the surface in air or due to contact with moisture, as well as due to differences in the state of the surface resulting from different fabrication processes. These differences affect the optical constant term in the applied linear model, which describes the thickness of the initial oxide layer formed on the surface. Its variation yields differences in the thickness obtained from different models of about 6 nm. Thus, it is necessary to choose one model, which can be used in further calculations. Consequently, model B was accepted since:

- It gave the best assessment of the initial layer thickness, compatible with the literature data [12,16],
- The surface preparation method decides the optical constant’s value for the substrate (incorrect determination of the refractive indices of bare metal substrates is one of the essential errors in ellipsometric measurements [16]). In our case, the best results were obtained for mechanically polished samples,
- Angle deviations were smaller than three degrees for all anodizing voltages. Other models did not fulfill this criterion.

However, it should be emphasized that the regression results obtained for model A do not differ statistically from model B; however, in the case of model A, for anodizing voltages of 30 and 35 volts, the angle deviations were greater than three degrees.

Our applied anodization procedure was proposed by Arsova et al. [12], and it is worth mentioning that the acceptance of optical constants from [12] and [16] did not give us reasonable results. One may suspect that the main reason for this outcome is the preparation of the surface and its state, which could be different in those experiments. While the refraction coefficient for Nb_2_O_5_ taken from [12] gave excellent fit, it did not work for Nb. It forced us to change the refraction coefficient for niobium and accept the one from [17].

Arsova et al. [12] determined the initial thickness of the oxide layer on Nb surface by extrapolation of experimentally determined linear dependence *d* = f(*U*). This gave an initial thickness of about 5 nm. However, one should be aware (and it was pointed out in earlier work [16]) that the surface preparation has substantial influence on this thickness. For example, after electroplating, this initial oxide layer is about 2.5 nm. This does not change the fact that neither the thickness of the initial layer nor the conditions of surface preparation may influence the linear dependence *d* = f(*U*). The real problem is the determination of the refraction coefficient for pure niobium. It was demonstrated by Arsova et al. [16] as well as in this work (Figure 3) that neither the initial thickness of the oxide layer nor the method of surface preparation have a decisive influence on the anodizing coefficient α. They affect substantially the free term b in the Equation (6):

(6) d=α·U+b

Having experimentally determined the dependence *d* = f(*U*), one can proceed with attempts to obtain the dielectric constant for Nb_2_O_5_. It can be derived from Equation (1) if the thickness of the oxide layer and the sample geometry are known and the capacitance *C* is measured. The EIS method is a very convenient experimental approach to achieve this aim. However, the success of its application depends heavily on the proper choice of the equivalent circuit, which represents the layer formed on niobium. The first condition we used was the number of time constants detected on Bode plots. The second time constant was visible for anodization potential greater than 30 V (oxide structure may change). Therefore, for higher potentials, the model of a single capacitor will not work. The coefficients characterizing the fit of the model to the data are chi-square χ^2^ and fitting errors for subsequent circuit elements were assessed using the software Z-View. Additionally, the results of measurements obtained for a single frequency are presented, which play the part of a “white box” containing only the capacitor. This kind of measurement with a single frequency can be often encountered in the literature [3,4,24]. Consequently, for voltages 0–30 V we chose the equivalent circuit shown in Figure 5a, while for higher voltages the circuit shown in Figure 5b will be appropriate.

The values of the capacitance obtained from EIS were combined with the thickness derived from ellipsometric measurements and described with Equation (1) to calculate the dielectric constant. The results of these calculations are gathered in Table 4. They differ from the values usually found in the literature [3,4,8,24], where ε is reported to be around 40 [3]. However, the electrolyte used in those experiments and the method of the oxide preparation are not specified. It is obvious that these factors must affect the dielectric constant. This situation is probably connected with the range of applied anodizing potentials, which did not exceed 10 V, as well as the thickness of the initial oxide layer, which is difficult to assess. The estimated error of this factor can be of the order of 10 nm. For anodization potential at zero volts, and assuming for this potential the thickness of the oxide equal to 3 nm, the relative error of the estimated dielectric constant is on the order of 300%. The increase in anodizing potential to 10 V brings about the thickening of the layer to 20–25 nm, which in turn reduces the relative error to about 40%. This implies that the results obtained for potentials in the range 15–30 V should possess an error less than 20%, which is a standard for commercial capacitors. Consequently, our obtained results can be considered to be compatible with the results of Cavigliasso et al. [8] and Young [4], who carried out their experiments for lower potentials. The observed differences can be attributed to problems with the determination of the thickness of the initial oxide layer. Results obtained for a single frequency may be treated as a reference point, assuming that the investigated system is an ideal capacitor. By applying adequately high frequency, the error resulting from a leakage can be neglected. This approach is, however, very sensitive to error resulting from frequency dispersion parameter *n* (value below 1 in CPE) [25].

## 5. Conclusions

Summarizing the results of this work, the following conclusions can be drawn:

- The thickness of the oxide layer on niobium surface can be precisely measured with the ellipsometric method when optical constants correspond to model B.
- The determined thickness vs. potential dependence has the form:
  *d* =2.14 (± 0.05) · *U* + 12.2 (± 1.7) (nm)
  in the range of applied potentials 0–50 V.
- The Nb sample was usually covered with the initial oxide layer, whose thickness was about 3 nm.
- In the potential range 0–30 V, the best fit to the EIS data can be obtained with the equivalent circuit shown in Figure 5a, which corresponds to a single oxide layer.
- As a result of combined ellipsometric and EIS methods, the best value of dielectric constant 93 ± 5 for the oxide Nb_2_O_5_ was obtained.
- Pits and holes may create two-layer structures responsible for the capacity collapse for anodization voltage above 30 V.

## Figures and Tables

**Table 1:** Ellipsometric optical constants for Nb and Nb_2_O_5_ for single layer model with measurement methodology.

Model Version	Nb Substrate Layer	Surface Preparation	Nb_2_O_5_ Layer	Surface Preparation and Measurement Conditions
A	2.850 - i∙2.990 [17]	Mechanically polished	2.270 - i∙0.0200 [16]	Mechanically polished / in situ
B	2.850 - i∙2.990 [17]	Mechanically polished	2.345 - i∙0.0120 [12]	Electropolished / ex situ
C	3.620 - i∙3.590 [16]	Electropolished	2.270 - i∙0.0200 [16]	Mechanically polished in situ
D	3.620 - i∙3.590 [16]	Electropolished	2.345 - i∙0.0120 [12]	Electropolished / ex situ

**Table 2:** Anodization coefficients and oxide thickness obtained from linear regression.

Model	Anodization Coefficient α (nm∙V^−1^)	Initial Oxide Layer (Calculated for Regression) (nm)	Natural Oxide Layer Measured Directly after Polishing (nm)	Coefficient of Determination (R^2^)
A	2.22 ± 0.05	13.73 ± 1.52	2.74	0.9961
B	2.14 ± 0.05	12.22 ± 1.69	2.66	0.9949
C	2.22 ± 0.06	19.15 ± 2.91	8.51	0.9939
D	2.13 ± 0.08	17.46 ± 2.35	8.30	0.9900

**Table 3:** Values for oxide capacitance *C*_x_ and parameter *n* (in all cases *C* in µF∙cm^−2^).

Anodization Voltage	Single-Layer Model		Double-Layer Model				Single Frequency
	*C*	*n*	*C*_1_	*n*_1_	*C*_2_	*n*_2_	*C*
0	66.6	0.9534					44.92
5	9.29	0.9647					6.81
10	5.07	0.9306					2.69
15	3.47	0.9035					1.92
20	2.07	0.9391					1.32
25	1.53	0.9491					1.01
30	1.29	0.9486					0.76
35			5.01	0.8889	126.4	0.6942	1.39
40			8.32	0.8822	382.2	0.5300	2.95
45			6.52	0.8175	11.7	0.8380	2.38
50			2.08	0.8851	5.6	0.9954	0.68

**Table 4:** Values of oxide dielectric constant ε_r_ for different measurements capacity method.

Voltage *U* (V)	0	5	10	15	20	25	30	35	40	45	50
A: Single frequency measurement	76	131	51	50	42	39	34	71	170	152	48
B: EIS single layer model	114	131	103	93	68	117	69				

**Table 5:** Anodization coefficient α for niobium oxidation.

Anodization Coefficient α (nm V^−1^)	Voltage/Potential Range (V)	Electrolyte	Comments	Work
2.22 ± 0.06	5–50	1M H_2_SO_4_	Oxide thickness measured using ellipsometry	This work
2.35 ± 0.06	0–8	0.5 M H_2_SO_4_	Oxide thickness calculated from the current flow	[8]
2.15 ± 0.05		1 M HNO_3_		
1.98 ± 0.05		1 M H_3_PO_4_		
2.40 ± 0.05		1 M NaOH		
2.35 ± 0.06		1M H_2_SO_4_		
2.26 ± no data	0–120	5.0 w/v% citric acid	Oxide thickness measured using ellipsometry	[12]
2.35 ± 0.03	10–100	0.5 M H_2_SO_4_	Oxide thickness measured using ellipsometry (data obtained from the plot)	[13]

## Data Availability

Not applicable.
